# Preparation of hydroxyethyl cellulose/ mangiferin edible films and their antimicrobial properties

**DOI:** 10.1186/s13065-022-00907-w

**Published:** 2022-12-12

**Authors:** Hebat-Allah S. Tohamy, Mohamed El-Sakhawy, Hossam M. El-Masry, Ibrahim A. Saleh, Mona M. AbdelMohsen

**Affiliations:** 1 Cellulose and Paper Department, National Research Centre, 12622 Cairo, Egypt; 2 Chemistry of Natural Microbial Products Department, National Research Centre, 33 El Buhouth Street, Dokki, P.O.12622, Cairo, Egypt; 3 Chemistry of Medicinal Plants Department, National Research Centre, 33 El-Bohouth St., Former ElTahrir St, Dokki, P.O.12622, Giza, Egypt

**Keywords:** Hydroxyethyl cellulose, Mangiferin, Edible films, Edible films, Packaging

## Abstract

In this study, we have used hydroxyethyl cellulose (HEC) to prepare antimicrobial films for multipurpose applications. Using HEC gives mangiferin powder (M) mechanical properties, while mangiferin powder gives HEC antimicrobial activities. Various concentrations of M (2.5, 5 and 10% wt/vol) were added to HEC to enhance the antimicrobial ability of HEC/M films. The results showed that 10% (wt/vol) was the optimum concentration to accomplish the antimicrobial activity. Various analyses were performed to study the prepared films’ physical, chemical, mechanical, and antimicrobial properties.

## Introduction

New environmentally friendly materials with tremendous potential and healthy functional features are in increasing demand. Biopolymeric materials based on natural components have recently seen an increase in demand due to their wide range of use [1]. The availability, degradability, biocompatibility, and nontoxicity of these biopolymers make them a good alternative to synthetic ones in food packaging [2–4].

The US Food and Drug Administration has approved hydroxyethyl cellulose (HEC), a nontoxic cellulose ether derivative that is non-ionic, biocompatible, tasteless, and hydrophilic [5]. It has a variety of uses, including coating, biomedical, pharmaceutical, and food items [6]. In addition, it has adhesion and film-forming properties, making it suitable for surface treatment and food packaging for preserving the quality of coated products, suppressing infections, and extending the shelf life of many fresh food species [7, 8].

Mango (*Mangifera Indica*) is a commercially significant fruit with Egypt’s leading cultivar. It is one of the most popular edible fruits and one of several countries’ most important commercial crops. Mango, like other plants, has bioactive chemicals that have been proven to have health advantages [9]. Fruit and vegetable wastes have been evaluated as potential bioremediation agents due to global concerns about utilizing biological solid waste. Food processing and agriculture companies generate fruit and vegetable wastes in large quantities. It can be employed as a low-cost, high-efficiency source of primary chemical constituents with varied functional groups and hence a wide range of biological activities [10]. Mangiferin is a bioactive component isolated from mango leaves, bark, peels, and kernels. It is a C glucosyl xanthone with the formula 1,3,6,7 tetrahydroxy xanthone–C 2-β D glucoside. Mangiferin has been demonstrated to have several positive health effects and is a viable candidate for further study and application. Mangiferin research has risen as pharmacology, and molecular biology offer more information for the design and development of mangiferin as a clinical treatment. It is abundant in dried mango peel, which is produced in huge amounts by the food processing and agriculture industries to further transform this fruit peel waste into a valuable product [11].

This study aims to prepare antimicrobial films using Hydroxyethyl cellulose (HEC) loaded with mangiferin powder (M) isolated from mango waste peel extracted with the eco-friendly microwave-assisted extraction (MAE) technique to get a bio-based film for food packaging applications. Consequently, the environmental problems at all levels were decreased. The films were characterized by Fourier transform infrared spectroscopy (FT-IR), X-Ray diffraction (XRD), and Scanning electron microscope (SEM) analysis. Besides, mechanical and wettability properties were examined. Finnaly, the film’s antimicrobial activity was tested against Gram-positive Staphylococcus aureus (*S. aureus*) and Gram-negative Escherichia coli (*E. coli*) bacteria. From discarded wates a bioactive mangiferin powder was extracted by a simple green method and utilized for antimicrobial films preparation.

## Experimental

### Materials

All materials and reagents were applied without further purification.

### Plant material and isolation of mangiferin

Mango fruits (*Mangifera indica L.*) are a national seasonal crop in Egypt which is collected every year in summer. The fruits were collected from an authorized productive farm in El- Bihara governorate and identified at the National Research Centre herbarium following national institutional guidelines and regulations. After consuming the fruits, the peels were washed several times with tap water and then allowed to dry in an oven at 40 ºC. A voucher sample was kept at the herbarium of the national research center (cultivated section).

The dried peel was crushed into powder and then extracted with 90% methanol using the microwave-assisted extraction (MAE) technique [12]. The methanol extract was concentrated under reduced pressure using a rotary evaporator. The slurry extract was dissolved in the least amount of 50% methanol, kept in the refrigerator overnight, then filtered. Using the liquid–liquid partitioning method, the extract was fractionated successively till complete exhaustion with petroleum ether, methylene chloride, ethyl acetate, and *n-*butanol (3 consecutive times for each solvent). The resulting portions of each solvent were evaporated to dryness. The concentrated mother liquor afforded a brown precipitate upon evaporation of the ethyl acetate fraction. This residue was washed with petroleum ether, methylene chloride, then methanol. Then, an aliquot of the residue was dissolved in an appropriate solvent and screened with TLC using percolated silica gel plates GF_254_ and different solvent systems, including methylene chloride: methanol (9:1), ethyl acetate: methanol (9.5:0.5) and ethyl acetate:formic acid:acetic acid:water (10:1.5:1.5:2.5). After spraying the plates with NA reagent, it showed the main spot with yellow color.

For further purification, the residue was applied to column chromatography using Sephadex LH 20. Methanol (95%) was used as the eluting solvent to isolate a pure compound as a yellowish-white powder [13].

### Preparation of HEC/Mangiferin edible films

1 g of HEC was dissolved in 20 ml H_2_O to form a gel solution. The gel was ultrasonicated with different ratios of M, i.e., 2.5, 5, and 10%, denoted as HECM1, HECM2, and HECM3, and then poured separately onto a Teflon plate and dried at 45 °C to attain a film of constant weight [14].

### Fourier-transform infrared spectroscopy (FT-IR)

Fourier-transform infrared spectra were collected employing Mattson 5000 spectrometer (Unicam, United Kingdom) using the KBr disk method. From the IR spectra, the mean hydrogen bond strengths (MHBS) were determined according to the relation:

$$MHBS = \frac{AOH}{ACH}$$

### Atomic absorption

Atomic absorption Perkin Elmer 3110 (United States) was employed to quantify the amount of metal ions.

### X-ray diffraction

The crystallinity was studied by X-ray powder diffraction. The diffraction patterns were measured by Bruker D-8 Advance X-ray diffractometer (Germany), applying a 40 kV voltage and a 40 mA current employing copper (K*α*) radiation (1.5406 Å).

$$Cr.l(\% ) = \frac{{Sc}}{{St}} \times 100,$$

$$\Delta {\rm{Cr}}.l(\% ) = \frac{{Cr.l\,of\,modified\,sample - Cr.l\,of\,unmodified\,sample}}{{Cr.l\,of\,modified\,sample}}{\rm{ \times }}100,$$

where Sc = area of the crystalline domain, St = area of the total domain, ΔCrI (%) = change of crystallinity.

*d* spacing (thickness) was calculated using Bragg’s law, and the crystallite size can be calculated by using Scherrer’s equation:

$$d(nm) = \frac{\lambda }{{2sin\theta }}{\rm{ \times }}100$$

$$Crystal\,size(nm) = \frac{{0.9\lambda }}{{\beta Cos\theta }}{\rm{ \times }}100$$

where θ = Bragg’s angle in radians, λ = X-ray wavelength (0.1542 nm), and β & θ are full widths at half maxima and Bragg’s angle of the XRD peak, respectively [15–17].

### Mechanical test

The tensile properties of the fabric specimens were measured by Universal Testing Machine- LLOYD LR 10 k, England. The tensile strength (TS), Elongation at break (EB %), and Young’s Modulus (Y %) of the films, were determined according to ASTM D-638 at a crosshead speed of 5 mm/min. The samples were cut into blocks of 100 × 3 × 20 mm^3^ (longitudinal × radial × tangential). Five specimens were tested for each sample, and the average value was listed.

### Repeatability and reproducibility of the conducted tests/analysis

To avoid sources of error inherent in a given measurement, every experiment was repeated 3 times and all analysis and tests are the average of five samples with less than 5% variation.

## Results and discussion

**Fig. 1:**
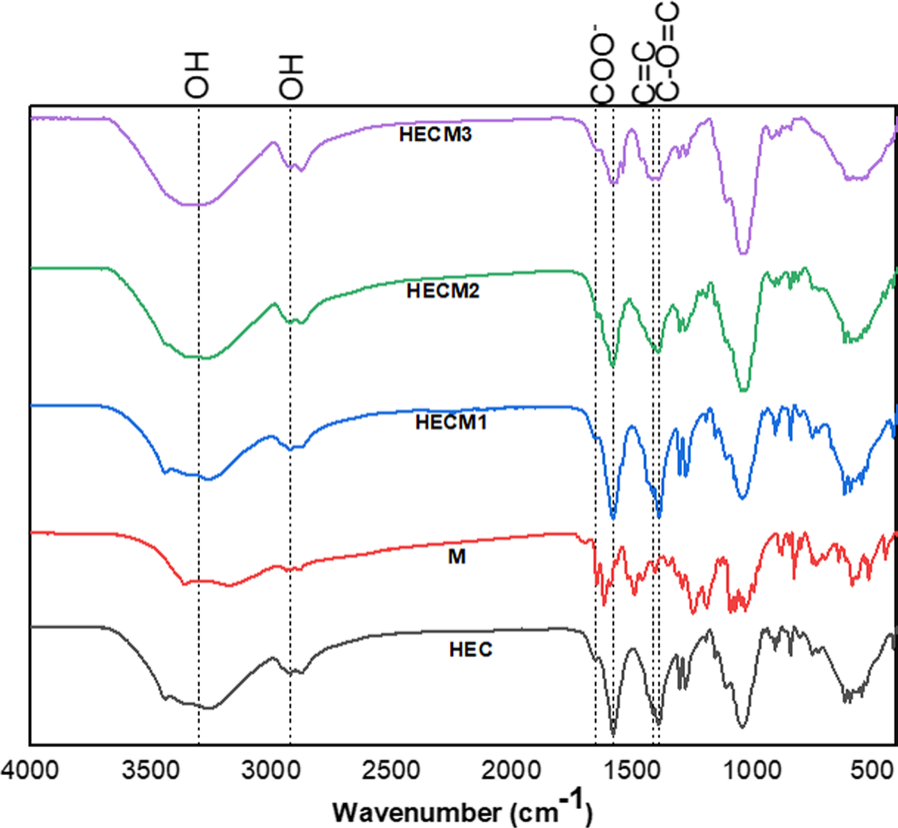
FTIR spectra of HEC, M, HECM1, HECM2, and HECM3, respectively

Figure 1 reveals the spectral analysis of HEC, M, HECM1, HECM2, and HECM3. The results showed that the characteristic functional bands assigned to HEC are OH-stretching ≈ 3262 cm^− 1^, anti-symmetric and symmetric CH_2_-stretching ≈ 2921 and 2875 cm^− 1^, anti-symmetric and symmetric stretching vibrations of COO^−^ ≈ 1653 and 1579 cm^− 1^, C=C ≈ 1415 cm^− 1^, C–O=C ≈ 1391 cm^− 1^, C–O–C ≈ 1110 cm^− 1^, C–O ≈ 1044 cm^− 1^ [16–18]. On the other hand, FTIR of M showed that OH-stretching ≈ 3171 cm^− 1^, CH_2_-stretching ≈ 2916 cm^− 1^, C=O in aldehyde and ketone ≈ 1652, 1621 cm^− 1^, and C=C ≈ 1490 cm^− 1^ [19]. Furthermore, the grafting of HEC was marked by increased intensity of the characteristic peaks of oxygen functionalities (ν_O–H_, and ν_C=O_). Since the band at 2921 cm^− 1^ is attributed to C–H stretching, this band can be used as an internal standard to determine the relative absorbance (RA) [20]. The RAs of the O–H were 0.82, 0.93, 0.93, and 0.94, while the RAs of the C=O were 0.67, 0.85, 0.93, and 0.97 for HEC, HECM1, HECM2, and HECM3, respectively. It is clear that the RAs of the O–H and C=O groups enormously increased by increasing the concentration of M compared to that of HEC.

The increase in crystallinity )LOI) and H-bonding (MHBS) of HECM3, as shown in Table 1, might be due to the decomposition of the amorphous parts of HEC during modification [20].

**Table 1:** The empirical crystallinity index (LOI) and mean H-bond strength (MHBS) of different samples

Sample	LOI (A_1425_/A_900_)	MHBS (A_OH_/A_CH_)
HEC	1.07	0.87
M	1.05	0.96
HECM1	0.94	0.93
HECM2	1.07	0.93
HECM3	1.012	0.94

**Table 2:** The crystallinity of the prepared samples

Sample	Cr.l (%)	$\varDelta$ Cr.l(%)	d (nm)	Crystal size (nm)
HEC	17.44	–	41.78	5.66
M	18.49	–	36.32	1.97
HEC1	5.84	− 66.49	44.28	1.26
HEC2	17.25	− 1.09	29.28	6.30
HEC3	25.92	48.62	19.29	5.07

**Fig. 2:**
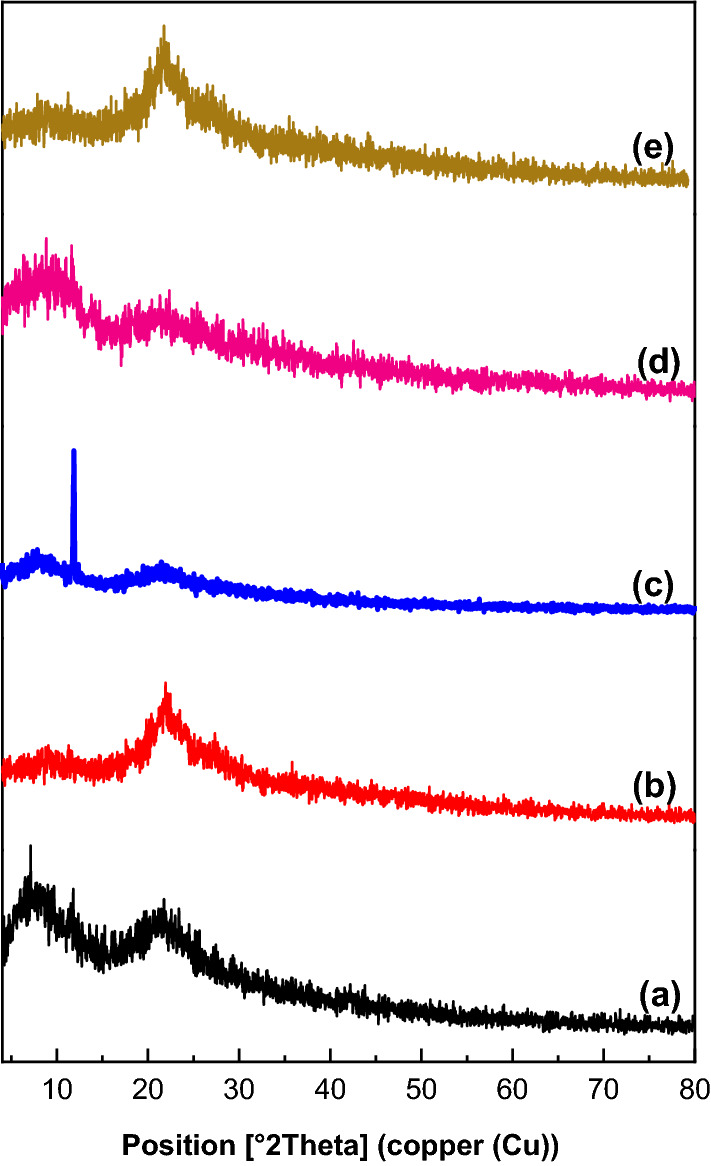
XRD analysis of **a** HEC, **b** M, **c** HECM1, **d** HECM2, and **e** HECM3, respectively

X-ray diffraction patterns of HEC, M, HECM1, HECM2, and HECM3 are presented in Fig. 2. Table 2 shows that the crystallinity indexes calculated for HEC, M, HECM1, HECM2, and HECM3 were 17.44, 18.49, 5.84, 17.25, and 25.92%, respectively. Cr.l (%) and d (nm) of the blended films (i.e., HECM1, HECM2, and HECM3) increased gradually due to the M concentration increase, which affects HEC film. The XRD crystallinity values agree with the MHBS values calculated from the IR spectra in the case of HECM1, HECM2, and HECM3.

**Fig. 3:**
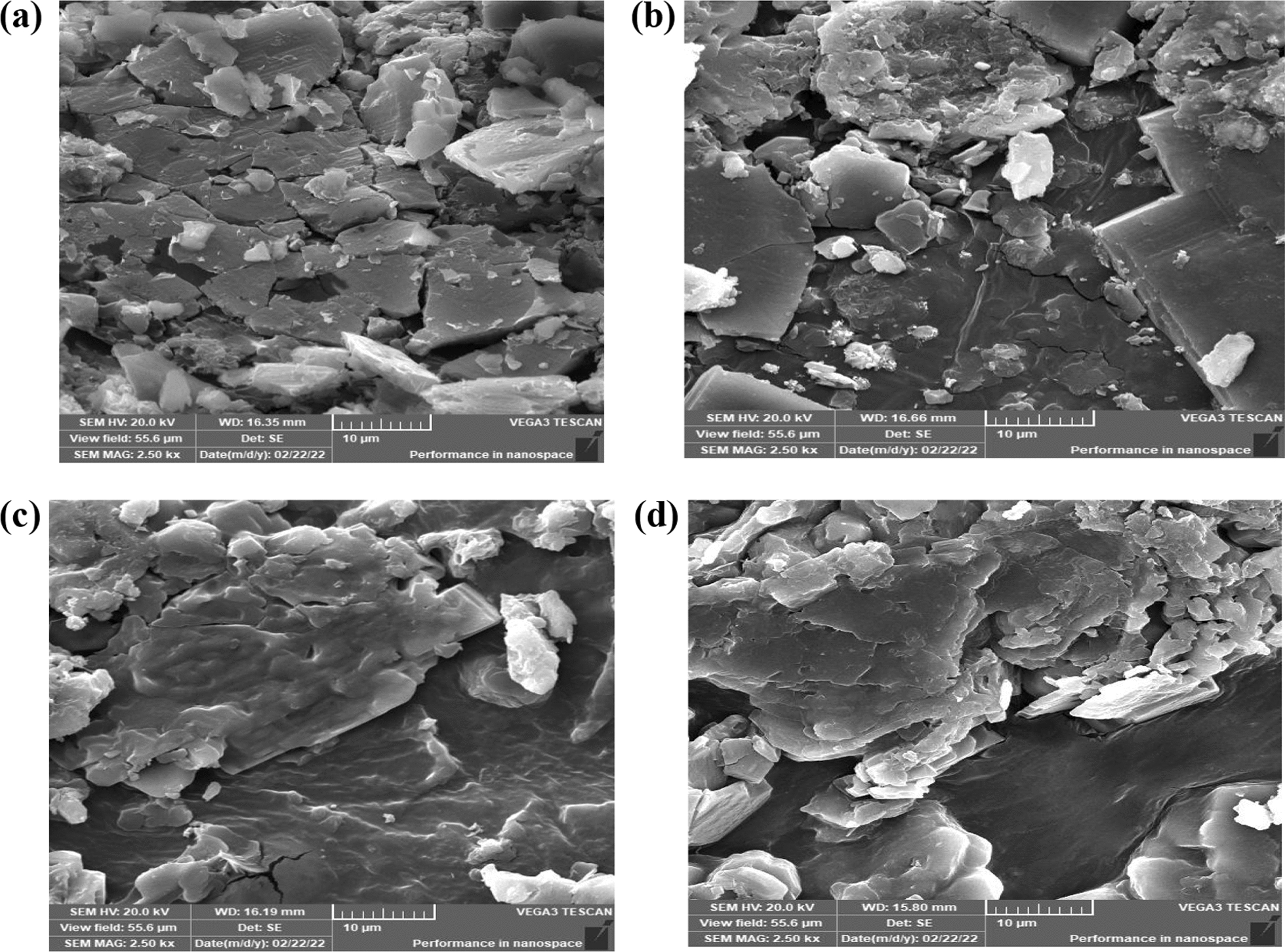
SEM images of** a** HEC,** c** HECM1,** d** HECM2 and** e** HECM3, respectively

The matrix’s structure, morphology, and homogeneity can affect the permeability of films. SEM was used to characterize the morphology of the composite films. SEM in Fig. 3 shows that HEC1, HEC2, and HEC3 appear more smooth and wavy than HEC blank. In addition, HEC3 is wavier than other modified films. The film’s porosity was calculated from the SEM figures and introduced in Table 3; the porosity was increased at higher M addition.

**Table 3:** Porosity (%) of the prepared samples

Sample	Porosity (%)
HEC	66.98
HEC1	55.74
HEC2	55.82
HEC3	99.81

Since tensile properties are essential for many applications, HEC matrix is blended with different M ratios. The tensile tests of HEC films with various loadings of M were investigated. Table 4 represents tensile strength (TS) at maximum load, elongation at break (EB%), and Young’s modulus (YM) of HEC, HECM1, HECM2, and HECM3, respectively.

According to the obtained results, the tensile properties of pristine HEC film have been remarkably affected by blending with varied M concentrations. Mechanical properties of pure HEC film, TS of 2.10 MPa, EB% of 147.69%, and YM of 2.98 MPa, are significantly changed after blending with 2.5, 5.0, and 10% of M. Upon loading of 10% M (i.e., HECM3); HEC film mechanical properties have primarily deteriorated. Percolation of a high concentration of M has destroyed the HEC polymer. YM is also affected by increasing the M content and reaches its maximum value of 7.39 MPa at HECM3 loading. YM enhancements can indicate homogeneous M dispersion through HEC matrix with HECM1, HECM2, and HECM3. These higher values observed for HECM1, HECM2, and HECM3 films in comparison with pristine HEC suggest the attenuation of the existing H-bonds between HEC by percolation of M and the establishment of new bonds between HEC and M in the film [21]. However, there was no conformity between the data of TS and MHBS. This was probably due to the type (intra- and inter-molecular), and degree of H-bonding.

**Table 4:** Mechanical properties of the prepared samples

Sample	Tensile strength (MPa)	Elongation at break (%)	Young’s modulus (MPa)
HEC	2.10	147.69	2.98
HECM1	1.43	127.13	5.14
HECM2	0.85	76.24	4.73
HECM3	0.56	94.98	7.39

From Table 4, an enhancement of YM values after M loading can be observed. YM increases with increasing M content from 2.5 to 10%. The remarkable increase in YM of the prepared films may be attributed to the formation of a more bonded network generated by adding M within the HEC matrix. This network results from strong electrostatic interactions and the H-bonding between M and HEC polymers. On the other hand, the EB% of films decreased considerably when loaded with M. The EB% of HECM1 does not change much from pristine HEC film. One possible reason can be attributed to the restricted motion of the HECM1 matrix by incorporating M in terms of the strong interactions between the fillers and the biopolymer matrix [21].

**Fig. 4:**
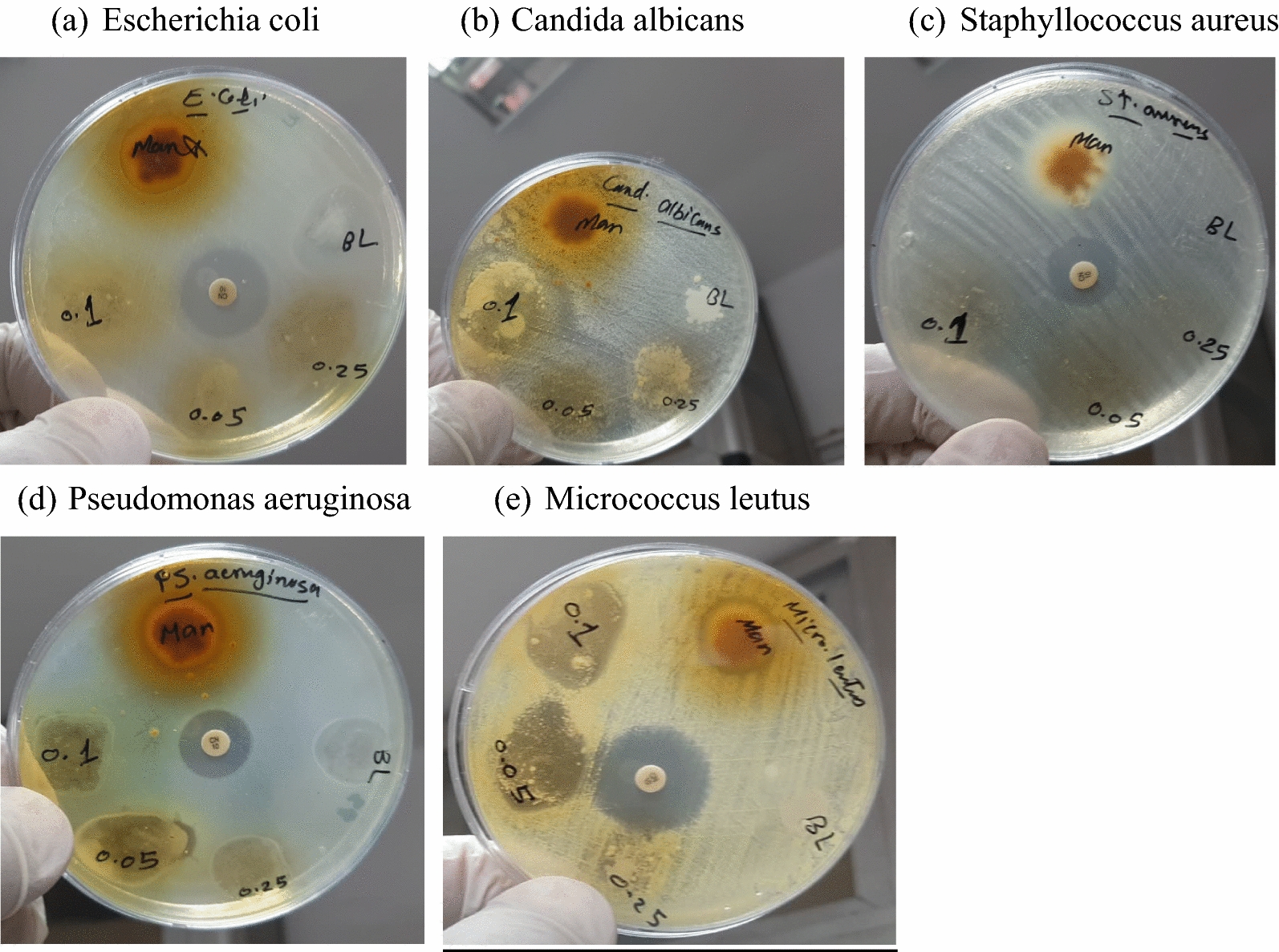
Antimicrobial activity of the prepared samples.

The most important medical discovery of the 20th century was the discovery of potent, relatively harmless antibacterial drugs. Antimicrobial agents’ modes of action against human, animal, and plant pathogens fall into one of four groups based on their action location. Inhibition of cell wall formation, protein synthesis, nucleic acid synthesis, and cell membrane integrity was among them [22]. Figure 4 shows that mango peel powder and its prepared samples have excellent antimicrobial activity against gram-positive bacteria (*Micrococcus leutus*, which shows a specific anti-gram-positive bactericidal spectrum). The prepared samples do not have any antibacterial and antifungal activity against two pathogenic gram-negative bacteria (*Escherichia coli* &* Pseudomonas aeruginosa*) and pathogenic fungal yeast (*Candida albicans*) respectively. In addition, they have modest antibacterial activity against gram-positive bacteria (*Staphylococcus aureus*).

The mode of action of mango peel powder and its prepared samples causes inhibition of bacterial cell walls, which is a mucopolysaccharide component called peptidoglycan (murein). So many actions take place toward gram-positive such as inhibition cell wall biosynthesis, inhibition biosynthesis of proteins, disrupt membranes, inhibition nucleic acid synthesis, mycobacterial adenosine triphosphate (ATP) synthase inhibitor, interact directly with PBPs and inhibit trans peptidase activity. These large molecules bind to the peptide chain of peptidoglycan subunits, blocking transglycosylation and transpeptidation and blocking the transport of peptidoglycan subunits across the cytoplasmic membrane. All these actions lead to the bactericidal effect of the sample (mango peel powder and its prepared films) against gram-positive bacteria (*Micrococcus leutus*). The effect of these samples (mango peel powder and its prepared films) on the cell wall of gram-positive is due to the presence of M powder, which enters inside the bacterial cell and ceases many cascades of action through inhibition of nucleic acid synthesis, which led to protein synthesis inhibition and followed the inhibition of all other bacterial metabolism activities. Also, they have this antimicrobial activity against pathogenic gram-positive bacteria *Staphylococcus aureus*, which is the skin-infected strain that causes many skin infections such as abscesses, wound infections, burning infections, and other skin infections, so the mango peel powder and its prepared samples, give a wide range of many application, such wound coatings, pharmaceutical and food packing.

Finally, the prepared samples have excellent antimicrobial activity toward gram-positive bacteria (*Micrococcus leutus*), which was improved by adding mango peel powder. Furthermore, antimicrobial activity was enhanced from 0.0 to 18.0 mm inhibition zone range, finally showing a promising specific anti-gram positive bacterial agent. Mangiferin is rich with a phenolic compound resemple to curcumin, and improved the antimicrobial properties of HEC films on the same way of cellulose, chitosan and collagen improvement by curcumin to fit wound healing applications [23]. Acutallly, chitosan shows some more prounnced results owing to its natural positively charge. It was proven that wound dressings based on natural polymer such as functionalized cellulose have an appropriate properties such as anti-adhesion, antimicrobial and skin reconstruction properties that can accelerate wound healing and inhibt eliminate microbial infections [24].

## Conclusion

HEC films were blended with M by ultrasonication. IR shows that the relative absorbance of the O–H and C=O groups enormously increased by increasing the concentration of M compared to that of HEC. X-ray diffraction patterns show that the crystallinity indexes increased gradually due to the increase in M concentration. SEM analysis shows that the porosity of HECM3 (99.81%) is enhanced compared to neat HEC (66.98%) due to the presence of M powder.

The blended HEC/M films have higher YM than neat HEC film and reaches its maximum value of 7.39 MPa at HECM3 loading, suggesting that the M were well dispersed within the HEC matrix, acting as an interpenetrated network, thus preventing water absorption by films when exposed to moisture. In addition, HEC films blended with M give mechanical properties to M powder while deteriorate HEC properties. In contrast, M powder offers antimicrobial properties to HEC due to their difference in aspect ratio. M powder enters inside the bacterial cell and ceases many cascades of action and followed inhibition of all other bacterial metabolism activities. Based on the obtained results of antimicrobial properties, loading with 10% M (i.e., HECM3) was chosen as the optimum condition for the film development.

## Data Availability

All data generated or analysed during this study are included in this published article.
